# Transcription activity hot spot, is it real or an artifact?

**DOI:** 10.1186/1753-6561-1-s1-s94

**Published:** 2007-12-18

**Authors:** Shuang Wang, Tian Zheng, Yuanjia Wang

**Affiliations:** 1 Department of Biostatistics, Mailman School of Public Health, Columbia University, 722 West 168^th ^Street, New York, New York 10032, USA; 2 Department of Statistics, Columbia University, 1255 Amsterdam Avenue, New York, New York 10027, USA

## Abstract

Transcription activity 'hot spots', defined as chromosome regions that contain more expression quantitative trait loci than would have been expected by chance, have been frequently detected both in humans and in model organisms. It has been common to consider the existence of hot spots as evidence for master regulation of gene expression. However, hot spots could also simply be due to highly correlated gene expressions or linkage disequilibrium and do not truly represent master regulators. A recent simulation study using real human gene expression data but simulated random single-nucleotide polymorphism genotypes showed patterns of clustering of expression quantitative trait loci that resemble those in actual studies [Perez-Enciso: *Genetics *2004, **166**: 547–554.]. In this study, to assess the credibility of transcription activity hot spots, we conducted genetic analyses on gene expressions provided by Genetic Analysis Workshop 15 Problem 1.

## Background

First pinpointed by Schadt et al. [1], expression quantitative trait loci (eQTL) 'hot spots', i.e., transcription activity hot spots, defined as chromosome regions that contain more eQTL than would have been expected by chance, have been points of research interest in almost all studies that search for genetic regulators for gene expression. Hot spots of gene regulation are most prominent in yeast [1,2], where eight have been detected. Hot spots have also been reported in differentiating xylem of a eucalyptus hybrid [3], mice [1], humans [4], and other organisms. Zheng et al. [5] observed hot spots harboring important breast cancer genes.

There are several interpretations of the existence of eQTL hotspots. The most common one states that hot spots could be due to some common regulatory elements that regulate transcription levels of a group of genes. Other interpretations are that eQTL hotspots represent gene-rich regions, or simply reflect the clustering of spurious QTLs from highly correlated expression levels, or from linkage disequilibrium (LD). A more recent study with expression data from two human genes with simulated single-nucleotide polymorphism (SNP) genotypes that are independent of the expression levels showed patterns of clustering of eQTL that resemble those published in human studies [6]. The observed enrichment was not random but neither was it caused by a putative mutation with a regulator effect, as all eQTL detected by design were false positives. The author concluded that the evidence of eQTL hotspots should be carefully evaluated and cautiously interpreted, and statistical analysis usually cannot distinguish between correlation and causation.

In this study, we aimed to assess and better understand features of transcription activity hot spots. We conducted a total of 3554 genome-wide linkage scans with 2819 autosomal SNPs on 3554 gene expression profiles. We found that high correlation between expression phenotypes might be a major source of contribution to the existence of hot spots. However, if a group of expression phenotypes are not correlated but are detected as transcription hotspots, the results might be more reliable and might represent a group of truly commonly regulated genes.

## Methods

### Centre d'Etude du Polymorphisme Humain (CEPH) samples

Based on 14 CEPH Utah families with 194 individuals, Genetic Analysis Workshop 15 (GAW15) Problem 1 provided 3554 gene expression profiles and 2882 SNPs across the genome (we used 2819 autosomal SNPs in the analyses), together with the physical map. Sex-specific genetic maps were provided by Sung et al. [7] and were used in the analyses.

### Linkage analysis

Genome-wide regression-based multipoint linkage analysis with quantitative traits was conducted with *merlin-regress *in MERLIN [8]. Merlin-regress determines evidence for linkage at each SNP based on a regression of estimated identity-by-descent (IBD) sharing between relative pairs on the squared sums and squared differences of trait values of the relative pairs [9]. Narrow-sense trait heritability was first estimated in MERLIN. The error-checking algorithm implemented in MERLIN was applied, and erroneous genotypes were excluded with command *pedwipe *before the linkage analysis.

### eQTL hotspots detection

To assess the clustering pattern of eQTL, we divided the autosomal genome into *N*_*B *_number of bins, each containing a fixed number of consecutive SNPs and with a smaller bin at the end of each chromosome. We then counted the number of genes with significant eQTLs in each bin. One 'hit' was counted for an expression phenotype if one or more SNPs within this bin were significant for the expression phenotype. The total number of hits, *N*_*H*_, along the autosomal genome can be defined this way. We hypothesized that if there was no enrichment in eQTL clustering, *N*_*H *_would be distributed randomly across the *N*_*B *_bins, thus the number of hits per bin will follow a Poisson distribution, with mean *N*_*H*_/*N*_*B*_. The significance of eQTL enrichment within each bin was therefore assessed using the Poisson distribution, and a Bonferroni correction was applied to account for the fact that *N*_*B *_tests were conducted.

To assess the reliability and credibility of the detected transcription activity hot spots, we conducted two analyses. First, we randomly removed one expression phenotype from a pair that has pair-wise correlation greater than a fixed value *ρ*, forming a subset of the gene expression that has pair-wise correlation smaller than *ρ*. More specifically, we first calculated all pair-wise correlations from the 3554 phenotypes and then randomly dropped one phenotype from the pairs that had pair-wise correlations greater than *ρ*. We then applied the same linkage analysis and hot spot detection procedure to the subset of the data with less correlated expression phenotypes. Second, we permuted the expression phenotypes within a family to generate a new data set that has no association between expression phenotypes and SNP genotypes and then applied the same linkage analysis and hot spot detection procedure.

## Results

We applied a stringent significance level in defining linkage signal and used a threshold of LOD > 5.3, corresponding to a point-wise *p*-value of < 3.9 × 10^-7^. The eQTL detected through this criterion has corresponding genome-wide threshold approximately 0.001. With this threshold applied to 3554 genome-wide scans, we observed 244 expression phenotypes that have evidence for linkage. The examination of regulators for the 244 expression phenotypes shows that gene-expression QTL are clustered, i.e., there are some transcription activity hot spots that contain more significant eQTL than would have been expected by chance across the created bins along the autosomal genome.

**Figure 1:**
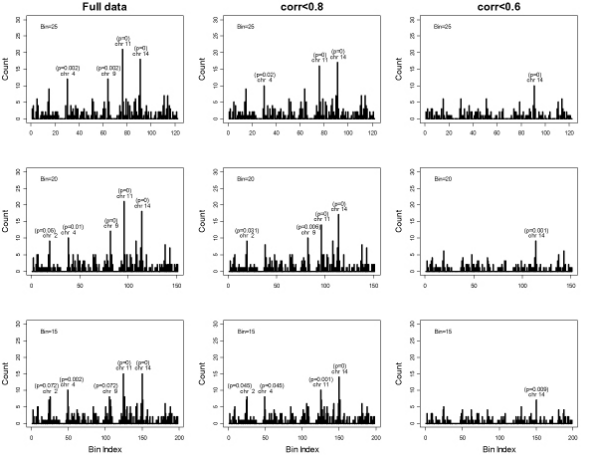
**eQTL cluster with different bin sizes andcorrelation levels**. LOD > 5.3. Only significant hot spots are highlighted with adjusted *p*-values.

To examine the effect of bin size on hot spot detection, we considered bins with 25, 20, and 15 consecutive SNPs (Figure 1). This yielded 122, 151, and 198 total bins, with each bin covering about 21.9 cM, 17.7 cM, and 13.5 cM. There are in total 305, 316, and 333 hits defined with the three different bin sizes, respectively. With a bin size of 25 SNPs, four significant hot spots were identified, where 21 phenotypes were mapped to one bin on chromosome 14, 18 phenotypes were mapped to one bin on chromosome 11, 12 phenotypes were mapped to one bin on chromosome 4, and 12 phenotypes were mapped to one bin on chromosome 9. If regulators for expression phenotypes were distributed randomly across the 122 bins, the probability of observing ten or more hits per bin would be less than 0.04 based on the Poisson distribution after Bonferroni correction. Similarly, with a bin size of 20 SNPs, besides the same four significant hot spots (bins of the detected hot spots with different sizes overlapped with each other) detected with a bin size of 25, one more significant hotspot on chromosome 2 was identified. With a bin size of 15 SNPs, the same three hot spots found for bin sizes of 20 and 25 on chromosomes 11, 14, and 4 were again detected. These results suggest that the current bin sizes considered do not influence the formation of hot spots dramatically.

To examine whether the hot spot is partially due to high correlation among expression phenotypes, we chose two thresholds and created two subsets by randomly removing one expression phenotype within a pair that has pair-wise correlation greater than 0.8, or randomly removing one expression phenotype within a pair that has pair-wise correlation greater than 0.6.

The two random subsets of expression phenotypes that either had pair-wise correlation smaller than 0.8 or had pair-wise correlation smaller than 0.6 resulted in retention of 3326 or 1754 expression phenotypes out of total 3554 expression phenotypes. Among the phenotypes retained, there were 227 and 131 expression phenotypes with evidence for linkage. These results are summarized in Table 1. The examination of the hot spots from the subset with pair-wise correlation smaller than 0.6 suggests that the high correlation between expression phenotypes might be one major source of the existence of eQTL clustering. For all three bin sizes considered, for the subset with pair-wise correlation smaller than 0.8, most of the hotspots identified with the full data set were preserved. While for the subset with pair-wise correlation smaller than 0.6, only the hotspot on chromosome 14 was still significant. Within this hot spot, there are eight, nine, and seven expression phenotypes mapped with bin sizes 25, 20, and 15, respectively. This may suggest that there is stronger evidence for the significant hot spot on chromosome 14 with eight expression phenotypes (bin size 25) to be a master regulator than original significant hotspot with 18 (bin size 25) expression phenotypes. Note that for the mapped 18 expression phenotypes on chromosome 14 with the full data, all significant linkage represent putative *trans *regulators.

**Table 1:** Summary of results from different bin sizes and different correlation thresholds

	Bin size^a^		
	
	25	20	15
Number of bins defined	122	151	198
Bin length (cM)	21.9	17.7	13.5
Full data (*n *= 3554)^b ^(No.^c ^sig. phenotypes = 244)			
Number of hits defined	305	316	333
No. sig. hot spots	4	5	4
corr < 0.8 (*n *= 3326)^b ^(No.^c^sig. phenotypes = 227)			
Number of hits defined	283	290	307
No. sig. hot spots	3	4	4
corr < 0.6 (*n *= 1754)^b ^(No.^c ^sig. phenotypes = 131)			
Number of hits defined	173	176	188
No. sig. hot spots	1	1	1

^a^number of consecutive SNPs

^b^total number of expression phenotype in the random generated subset

^c^number of expression phenotypes with evidence of linkage in the random generated subset

Based on the results from bin size 25, we further examined the functions of the 18 genes in the hot spot on chromosome 14. We noted that the 8 out of 18 genes in the hot spot from the subset of expression phenotypes with pair-wise correlation smaller than 0.6 expressed "molecular binding" more specifically (Table 2). Two explanations are possible here, and results should be interpreted with care. First, for the genes that are indeed commonly regulated and are also highly correlated, when a subset of genes is removed, the true hot spot signal might be weakened. On the contrary, if the genes are not truly commonly regulated but are highly correlated, after removing a subset of genes, the hot spots that remain significant might truly represent master regulation.

**Table 2:** Biological properties of the clustered expression phenotypes within the hotspot on chromosome 14

Gene	Location	Gene ontology molecular function	Gene ontology biological process
***DDX24***^a^	**chr14q32.13**	**nucleotide binding**	**RNA metabolism**
***FDPS***	**chr1q22**	**transferase activity**	**cholesterol biosynthesis**
***TRAM2***	**chr6p12.2**	**NA**	**protein targeting**
***AP3B1***	**chr5q14.1**	**binding**	**intracellular protein transport**
***PDIA3***	**chr1q21.1**	**protein disulfide isomerase activity**	**electron transport**
***SMARCB1***	**chr22q11.23**	**protein binding**	**chromatin remodeling**
***CBARA1***	**chr10q22.1**	**calcium ion binding**	**defense response**
***RAP80***	**chr5q35.2**	**NA**	**transcription**
*GSTO1*	chr10q25.1	glutathione transferase activity	metabolism
*IGBP1*	chrXq13.1	protein phosphatase type 2A regulator activity	response to biotic stimulus
*LSM3*	chr3p25.1	RNA binding	nuclear mRNA splicing, via spliceosome
*INPP5A*	chr10q26.3	inositol phosphatase activity	cell communication
*SEC13L1*	chr3p25.3	NA	intracellular protein transport
*TXNDC*	chr14q22.1	electron transporter activity	DNA replication
*RPN2*	chr20q11.23	transferase activity	protein modification
*ATG5*	chr6q21	NA	autophagy
*NDUFB2*	chr7q34	NADH dehydrogenase activity	generation of precursor metabolites and energy
*ZA20D3*	chr15q25.1	DNA binding	NA

^a^Bold text indicates the subset of eight genes from less correlated expression phenotypes.

**Figure 2:**
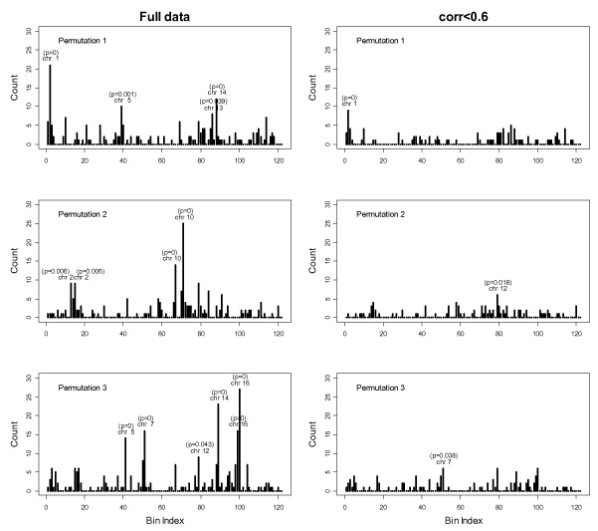
**Significant eQTL are clustered in the permuted data**. LOD > 5.3 and bin size = 25; only significant hot spots are highlighted with adjusted *p*-values.

Analysis of permuted data sets when no association exists between expression phenotypes and SNP genotypes also suggested clustering of eQTL, consistent with what was observed by Perez-Enciso [6]. Analysis of the subset of permuted data with pair-wise correlation smaller than 0.6 confirmed the above observation from the original data that high correlation between expression phenotypes might be a major source of the existence of hot spots. Results from three random permutations are presented in Figure 2. Note that for permutations 2 and 3, the most significant hot spot identified contain 25 and 27 gene expressions. However, neither hot spot is significant in the less correlated subset of the data. Only 4 out of 25 mapped gene expressions were preserved in the hot spot on chromosome 10 in permutation 2, and only 6 out of 27 mapped gene expressions were preserved in the hot spot on chromosome 16 in permutation 3. This suggests that the formation of the most significant hot spot here is mainly due to the high correlation between expression phenotypes, and the significant hot spot from the subset of less correlated data might suggest true master regulation. However, we should note that the hot spots here are, by construction, false positives.

## Discussion

Although it has been common to consider the existence of hot spots as evidence for master regulation of gene expression, we should always be more cautious in interpreting such results because the findings might be simply due to highly correlated gene expressions or linkage disequilibrium and do not truly represent master regulation. In this study, in order to assess the reliability and credibility of frequently detected transcription activity hot spots, we conducted two analyses on all 3554 gene expression phenotypes using GAW Problem 1 data. Note that no screen steps were applied to select a subset of gene expression profile. Although this may bring noise to the analysis, Huang et al. [10] suggested that gene expressions with very low heritability may show very high linkage signals. Further research and more careful selection procedures are definitely needed. We first created a subset of data with pair-wise correlation smaller than a fixed value, and then examined the existence of eQTL hot spots. The results suggest that two explanations are possible. First, if genes that are indeed commonly regulated and are also highly correlated, removing a subset of highly correlated genes might weaken the hot spot signal; second, for genes that are not commonly regulated but somehow are highly correlated, when we remove a subset of highly correlated genes, the hot spots that remain detected might truly represent master regulation. Results from permuted data both with and without highly correlated expression phenotypes confirm the above findings. Experimental results should always be interpreted with caution and more thorough analyses need to be conducted before reaching any firm conclusions.

## Competing interests

The author(s) declare that they have no competing interests.
